# Microwave Assisted Selective Hydrolysis of Polyamides from Multicomponent Carpet Waste

**DOI:** 10.1002/gch2.202000119

**Published:** 2021-05-05

**Authors:** Eva Bäckström, Karin Odelius, Minna Hakkarainen

**Affiliations:** ^1^ Department of Fibre and Polymer Technology School of Engineering Sciences in Chemistry Biotechnology and Health KTH Royal Institute of Technology Teknikringen 56–58 Stockholm SE‐100 44 Sweden

**Keywords:** carpet waste, microwave, polyamides, polypropylene selective hydrolysis, recycling

## Abstract

Selective hydrolysis of polyamide‐6 (PA‐6) and polyamide‐66 (PA‐66) from commercial multicomponent PA‐6/PA‐66/polypropylene (PP) carpet is demonstrated by a microwave‐assisted acid catalyzed hydrothermal process, yielding monomeric products and solid polypropylene residue. First, an effective method is established to chemically recycle neat PA‐6 and PA‐66 granules using microwave irradiation. The optimized, hydrochloric acid (HCl) catalyzed process leads to selective production of monomers, 6‐aminocaproic acid or adipic acid and hexamethylenediamine, after only 30 min. A piece of commercial carpet is then recycled using the same reaction conditions, but with the alteration of the reaction time from 1 to 6 h. The produced water‐soluble products and the remaining solid residue are carefully characterized, proving that the polyamide‐part of the carpet is selectively hydrolyzed into water‐soluble monomers and the polypropylene‐part remains as an unconverted solid that can be further used to produce recycled filaments containing the carpet residue and virgin polypropylene. The developed process opens the possibility to recycle multicomponent materials, such as carpets, through selective hydrolysis. It can also contribute to a circular economy, producing original monomers and materials ready for a new life‐cycle.

## Graphical abstract

A fast, efficient, and selective microwave‐assisted recycling of polyamide/polypropylene carpet waste is demonstrated. During this process, polyamide fibers are hydrolyzed back to monomers, while the polypropylene part can be collected as a solid residue. The method does not require a complicated separation step and the produced products are ready for further usage.

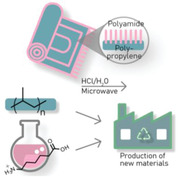

## 1 Introduction

Synthetic fibers are used in many applications, such as clothes and carpets. In 2014, 55 million tonnes of synthetic fibers were produced, compared to merely 69 thousand tonnes in 1950.^[^ ^1^, ^2^ ^]^ These synthetic fibers are non‐biodegradable, difficult to recycle, and they are made from petroleum resources, a non‐renewable feedstock.^[^ ^3^ ^]^ Consequently, the amount of carpet waste is large, causing environmental impact. Annually, 1.6 million tonnes of carpet waste is produced in the EU alone and most of this waste is placed in landfills or incinerated; only 3% of the waste is recycled.^[^ ^4^, ^5^ ^]^ An increasing number of countries are legislating against landfills, and incineration does not retain the intrinsic material value of the polymeric materials which is a waste of resources.^[^ ^6^ ^]^ Synthetic carpets usually consist of a multicomponent system, where several different polymeric materials constitute the fibers and backing, respectively. The advantage of the multicomponent system is the specific properties and performances of the different polymers. This is, however, a disadvantage when it comes to recycling, as sorting and separation become challenging and polymers are seldom miscible. They also have different characteristic behaviors such as solubility and processability.^[^ ^7^, ^8^ ^]^ This calls for new techniques to recycle carpet waste to respond to environmental and economic concerns and to generate valuable products from the waste.^[^ ^6^, ^9^, ^10^, ^11^, ^12^ ^]^

Many synthetic multicomponent carpets comprise of polyamide (PA) fibers (polyamide‐6 (PA‐6) and/or polyamide‐66 (PA‐66)) with a polypropylene (PP) backing, as well as an adhesive to ensure that the fibers are well connected to the backing. Additives such as dyes, repellents, and inorganic fillers (e.g., calcium carbonate, CaCO_3_) are also commonly added.^[^ ^8^, ^13^ ^]^ PA fibers are often chosen, because of their high melting points and high abrasion resistance.^[^ ^6^ ^]^ Typically, the PAs make up around 50% of the carpet mass, making recycling of the PA section a viable option.^[^ ^14^ ^]^ The recycling can take place through, for example, selective hydrolysis of the PA component present in the synthetic carpet.^[^ ^15^ ^]^

Chemical recycling converts waste into new building blocks such as monomers, oligomers or functional chemicals, that in turn can be utilized to synthesize new polymers or plasticizers.^[^ ^16^, ^17^ ^]^ In this way, the value of the material can be retained or in some cases, it can even increase.^[^ ^18^ ^]^ This is a potent option for PAs, since they can be depolymerized by hydrolysis of the amide bond, resulting in the cyclic monomer ε‐caprolactam^[^ ^19^ ^]^ or the linear monomer aminocaproic acid from polyamide‐6 and adipic acid and hexamethylenediamine from PA‐66. These studies show that high temperatures and pressures are effective tools for depolymerization.^[^ ^3^, ^5^, ^6^, ^20^ ^]^ Successful depolymerization of PA‐6 was also achieved by microwave assisted recycling under high temperatures and preasures. Concentrated phosphoric acid (C = 0.50 g mL^−1^) was for example used as a catalyst, leading to a product mixture containing 90% aminocaproic acid, the linear form of ε‐caprolactam during 12 minutes at 240 °C. This catalyst was chosen due the the high dipole moment of phosphoric acid, which makes it a good microwave absorbent.^[^ ^21^, ^22^ ^]^ In recent study, polyamides were depolymerized by utilizing hydrochloric acid as a catalyst. PA‐66 was completely converted to monomers after 30 min of microwave irradiation at 200 °C with a molar ratio of polyamide to hydrochloric acid of 1 to 1.25.^[^ ^23^ ^]^ Compared to reflux and autoclave reactions, the microwave‐assisted reaction was much faster. Our previous works also show that microwave‐assisted reactions can effectively depolymerize both biopolymers and synthetic polymers to functional chemicals and carbon materials for further generation of new materials.^[^ ^17^, ^21^, ^24^, ^25^, ^26^, ^27^ ^]^

The most common procedure to recycle PA/PP carpet waste is by first separating the two constituents.^[^ ^13^ ^]^ However, efforts have also been made to retrieve the polyamide monomers by pyrolysis and by steam treatment without separation of the constituents beforehand. For example, a PA‐6/PP blend was pyrolyzed to recover ε‐caprolactam.^[^ ^28^ ^]^ Steam has also been used to depolymerize the PA‐6 part of a PA‐6/PP carpet. The remaining carpet residue could be collected after the reaction.^[^ ^29^ ^]^ None of these studies did any detailed characterization on the solid residues, however, they show that high temperature and extended times were required to selectively recycle multicomponent carpet waste.

The aim of the current work was to utilize the effectiveness of microwave‐assisted reactions to achieve easy and fast chemical recycling of multicomponent carpet waste by selective hydrolysis of the PA part to monomers. Ideally this would leave the PP backing unaffected and ready to be re‐entered in the production cycle. Our hypothesis was that this methodology would circumvent the complicated separation step of polyamide from polypropylene, which would be valuable for recycling carpet waste, but also for recycling other multicomponent materials thereby contributing to a more circular economy.

## 2 Results and Discussion

### 2.1 Catalyst‐Free Microwave‐Assisted Recycling of PA‐6 and PA‐66 Granules

To develop an effective method to recycle multicomponent carpet waste, a methodology was first developed for the chemical recycling of pure PAs to their corresponding linear monomers. Water without catalyst was initially tested for its ability to hydrolyse PA‐6 and PA‐66 granules during microwave processing. Run times between 2 and 4 h at 200 °C were evaluated, but no water‐soluble products were detected under these conditions, Table S1, Supporting Information. The produced solid powders as well as the original PA granules were characterized by matrix‐assisted laser desorption ionisation‐mass spectrometry (MALDI‐MS), Figure S1, Supporting Information. As MALDI‐MS analysis can discriminate against higher molecular weights for samples with high dispersity, it was not possible to determine the molecular weights of the original PAs. Instead the low molecular weight fraction of the material was detected. Analysis of PA‐6 displayed peaks between 500 and 3000 m/z, while only low intensity peaks were detected in analysis of original PA‐66. After microwave assisted hydrolysis, peaks corresponding to oligomeric PAs as sodium adducts (m/z = 113n +18+23) for PA‐6 and (m/z = 226n+18+23) for PA‐66 were identified. 113 and 226 correspond to the molecular weights of the repeating units of PA‐6 and PA‐66; *n* is the number of repeating units, 18 corresponds to the molecular weight of the end groups, —H and —OH, and 23 is the molecular weight of sodium. The peaks with the highest intensity had an m/z ratio of ≈1000–1200 for both oligomeric PA‐6 and PA‐66. This shows that without catalyst, PAs can be hydrolyzed to a certain extent, but the time‐frames evaluated were not long enough to yield water‐soluble products. All of the spectra after 2 and 4 h of microwave assisted hydrolysis appeared quite similar. As described above, MALDI‐MS analysis can discriminate higher molecular weights, making it difficult to draw a definite conclusion concerning the effect of different hydrolysis times. The products were similar to those produced in a previous study, where PAs were refluxed in water and methanesulfonic acid.^[^ ^30^ ^]^

### 2.2 Acid Catalyst Selection for Microwave‐Assisted Recycling of PA‐6 and PA‐66 Granules

To catalyse the hydrolytic recycling of PA‐6 and PA‐66, aqueous solutions of hydrochloric acid (HCl), phosphoric acid (H_3_PO_4_), and sulfuric acid (H_2_SO_4_) as well as a solution of HCl in methanol (MeOH), all with a concentration of 0.1 g mL^−1^, were prepared and evaluated. The reaction time was set to 2 h and the reaction temperature was set to 200 °C, except for the HCl‐MeOH solution for which 150 °C was used because of the lower boiling point of MeOH compared to water. PA‐6 was selected for the initial testing.

#### 2.2.1 Identification of Water‐Soluble End‐Products

**Figure 1:**
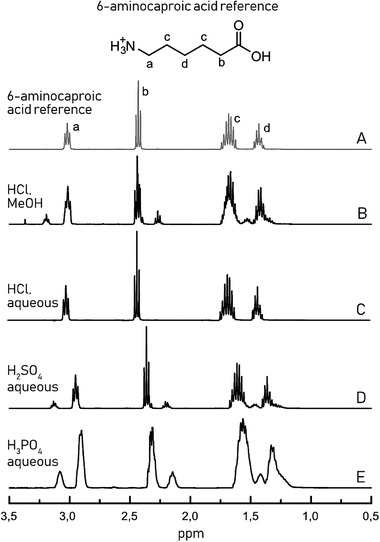
^1^H‐NMR spectra of the water‐or MeOH‐soluble hydrolysis products obtained after 2 h of microwave processing of PA‐6 using different catalysts at C = 0.1 g mL^−1^. A) Protonated 6‐aminocaproic acid included as a reference and the products obtained after heating in B) HCl–MeOH at 150 °C, C) aqueous HCl at 200 °C, D) aqueous H_2_SO_4_ at 200 °C, and E) aqueous H_3_PO_4_ at 200 °C.

After microwave recycling, the water‐ or MeOH‐soluble phases were collected, and the solvent was removed by rotary evaporation and drying in a vacuum oven. After drying, the obtained products were analysed by nuclear magnetic resonance spectroscopy (NMR). If complete hydrolysis to monomeric products is achieved, the expected end‐product is aminocaproic acid, the linear form of ε‐caprolactam.^[^ ^13^ ^]^ When HCl is used as a catalyst, the hydrochloride salt is formed as the final monomeric product.^[^ ^23^ ^]^ Protonated 6‐aminocaproic acid was therefore analysed as reference standard compound. The characteristic peaks in ^1^H‐NMR were identified at 3.02 ppm (a), 2.43 ppm (b), 1.68 ppm (c), and 1.43 ppm (d), **Figure** **1**A. The water‐ or MeOH‐soluble products obtained after microwave processing of PA‐6 with different catalysts can be seen in Figure 1B–E. Complete depolymerization to monomers was not achieved with H_2_SO_4_ and H_3_PO_4_ as catalysts within the set time frame (peaks indicating presence of oligomers can be seen at approximately 3.10 and 2.17 ppm) and the catalysts were difficult to remove after hydrolysis. Therefore, they were not considered for further experiments. The spectrum of the products obtained after microwave processing in MeOH with HCl as catalyst, displayed numerous products, indicating that oligomers instead of monomers were produced, or side‐reactions occurred. The peaks indicating oligomers can be seen at 3.20 and 2.27 ppm. If all the spectra are compared, the peaks shift slightly depending on which acid was used as a catalyst, due to small differences caused in the local environment. Aqueous HCl was chosen as a reaction medium for further experiments, since complete recycling to monomers was achieved and no side‐reactions occurred in this medium, with the ^1^H‐NMR spectrum matching well with the spectrum of the expected monomer protonated 6‐aminocaproic acid. The reason why HCl as a catalyst produced the best results is explained by its lower pKa value compared to the other acids, causing the scission of the amide bond to occur faster. The strong acid combined with high temperature and pressure in the microwave reactor enabled complete depolymerization.

### 2.3 The Effect of Acid Concentration and Time on Microwave Recycling of PA‐6 and PA‐66 Granules

After catalyst selection, the microwave recycling of PA‐6 and PA‐66 was evaluated at 200 °C with aqueous HCl as catalyst. Two catalyst concentrations, 0.1 g mL^−1^ and 0.01 g mL^−1^, were evaluated in depth by varying the reaction times from 30 min to 3 h. For exact reaction parameters and a summary of the obtained results, see **Table** **1**. The percentage of remaining solid material was calculated by Equation (1), where the remaining solid material (water‐insoluble) was compared to the original amount of PA.

**Table 1:** Summary of reaction conditions and type of products obtained after microwave recycling of PAs

Starting material	Time [h]	C HCl aq [g mL^−1^]	Remaining solid material [%]	Water soluble products
PA‐6	0.5	0.01	0	Yes, oligomeric
PA‐6	1	0.01	0	Yes, oligomeric
PA‐6	2	0.01	0	Yes, oligomeric
PA‐6	3	0.01	0	Yes, oligomeric
PA‐6	0.5	0.1	0	Yes, monomeric
PA‐6	1	0.1	0	Yes, monomeric
PA‐6	2	0.1	0	Yes, monomeric
PA‐6	3	0.1	0	Yes, monomeric
PA‐66	0.5	0.01	12.5	Yes, oligomeric
PA‐66	1	0.01	12.5	Yes, oligomeric
PA‐66	2	0.01	3.0	Yes, oligomeric
PA‐66	3	0.01	2.7	Yes, oligomeric
PA‐66	0.5	0.1	5.2	Yes, monomeric
PA‐66	1	0.1	16.6	Yes, monomeric
PA‐66	2	0.1	25.3	Yes, monomeric
PA‐66	3	0.1	35.1	Yes, monomeric

**Figure 2:**
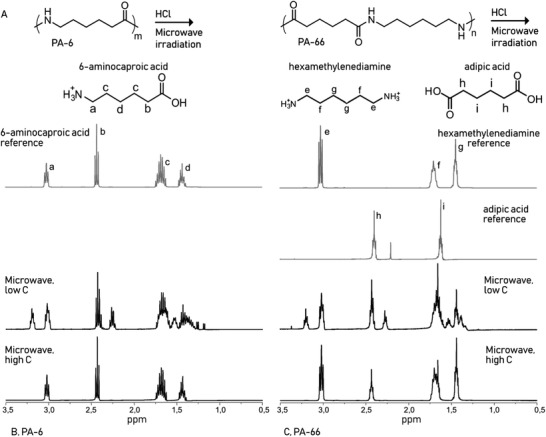
A) Schematic presentation of monomers produced by microwave‐assisted recycling of PA‐6 and PA‐66. ^1^H‐NMR spectra of the water‐soluble end products after 30 min of microwave processing: B) end products of PA‐6 recycling and C) end products of PA‐66 recycling. ^1^H‐NMR spectra of standard compounds corresponding to the expected end products are included in both cases for comparison.

After recycling of PA‐6 in the microwave at a low concentration of HCl (0.01 g mL^−1^), no insoluble products remained irrespective of recycling time. When PA‐66 was microwave recycled with the low concentration of HCl (0.01 g mL^−1^), the amount of solid product decreased after longer reaction time; see Table 1. The opposite results were seen at higher HCl concentration (0.1 g mL^−1^). The higher HCl concentration in the solution could possibly promote repolymerization or cause other reactions to take place during prolonged heating, generating non‐water soluble products.^[^ ^3^ ^]^ No solid products remained after microwave processing of PA‐6 under the different tested reaction conditions, Table 1. However, when low HCl concentration was utilized, a mixture of oligomers was obtained, while high HCl concentration led to formation of monomeric products. A simplified scheme for the chemical recycling reaction is illustrated in **Figure** **2**A. Some solid product remained after microwave processing of PA‐66 both with low and high HCl concentration. This is due to the higher melting temperature of PA‐66 and the longer alkyl segment between the amide bonds.^[^ ^23^ ^]^ Interestingly, the same phenomenon with oligomeric versus monomeric products was observed, depending on the concentration of HCl, that is, at higher HCl concentration (C = 0.1 g mL^−1^), water soluble monomers were formed from both PA‐6 and PA‐66, Figure 2.

#### 2.3.1 Identification of Water‐Soluble End‐Products

The water‐soluble products formed during microwave‐recycling match well with the spectra of the expected monomeric products: protonated 6‐aminocaproic acid (protons a (3.02 ppm), b (2.43 ppm), c (1.68 ppm), and d (1.43 ppm)) for PA‐6 (Figure 2) and protonated hexamethylenediamine (protons e (3.03 ppm), f (1.71 ppm), and g 1.45 ppm)) and adipic acid (protons h (2.40 ppm) and I (1.62 ppm)) for PA‐66. Hence, effective chemical recycling of PA‐6 and PA‐66 to original monomers was demonstrated (Figure 2C). The water‐soluble products formed after microwave recycling of PA‐6 and PA‐66 in 0.01 g mL^−1^ HCl are shown in Figure 2B. Peaks originating from oligomers can be seen at 3.19 and 2.26 ppm for PA‐6 and at 3.19 ppm and 2.27 ppm for PA‐66. To verify that the produced monomers could be used as building blocks for new polymers, the crude products were directly step‐growth polymerized. See Figure S2, Supporting Information, for MALDI‐MS analysis of the produced oligomers. PAs are relatively expensive polymers so chemical recycling and repolymerization is a good option.^[^ ^31^ ^]^

**Figure 3:**
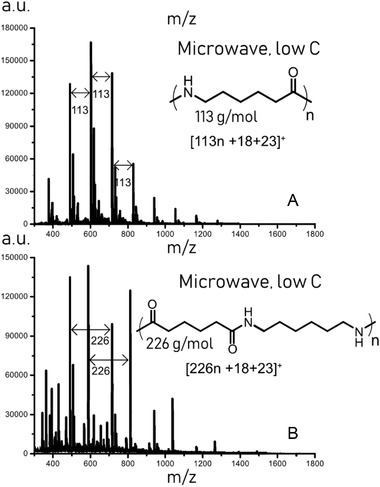
MALDI‐MS mass spectra of the water‐soluble oligomers formed after microwave processing of PA‐6 and PA‐66 for 30 min at an HCl concentration of 0.01 g mL^−1^. A) end‐products after microwave processing of PA‐6, B) end‐products after microwave processing of PA‐66.

The presence of oligomers was further confirmed by MALDI‐MS analysis, **Figure** **3**. The peaks corresponding to oligomeric PAs were identified as sodium adducts (m/z = 113n + 18+ 23) for PA‐6 and (m/z = 226n+18+ 23) for PA‐66.^[^ ^30^ ^]^ For the PA‐66 oligomers, two series of peaks can be seen. This is due to the repeating unit consisting of two different monomers, resulting in larger variety of possible oligomers and end‐group combinations.^[^ ^22^, ^32^ ^]^

### 2.4 Selective Microwave‐Assisted Hydrolysis of PAs in Carpet Waste

**Figure 4:**
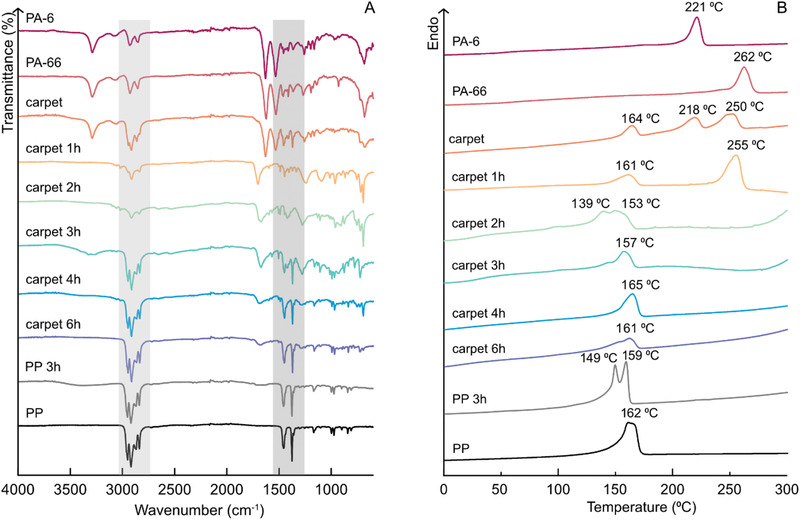
A) FTIR‐spectra of the remaining solid carpet material after different microwave recycling times, neat carpet and PA‐6, PA‐66, and PP references. B) DSC thermogram of the same samples.

To take the developed microwave recycling method for PAs to the next level, it was applied to a piece of commercial carpet. differential scanning calorimetry (DSC) and Fourier transform infrared resonance spectroscopy (FTIR) analysis of the carpet confirmed PA‐6, PA‐66, and PP as the main components in the carpet (**Figure** **4**). In addition to PA‐6, PA‐66, and PP, the carpet waste could contain, for example, adhesives and CaCO_3_ that could consume some of the HCl during the microwave recycling process. An attractive way to recycle this kind of product would be selective hydrolysis of the PA component back to monomers, which could be easily separated for further use, followed by recovery of the solid PP part. The PP part could be further used, for example, as a structural component in composites^[^ ^8^ ^]^ or be mechanically recycled. Since the PAs are hydrolysed back to monomers, the initial molecular weight of the PAs is not significant and the impurities (e.g., harmful flame retardants) and dirt in the carpet could be collected during product purification.

Here, the carpet was subjected to microwave recycling from 1 to 6 h at 200 °C and the utilized HCl concentration was 0.1 g mL^−1^, since this higher concentration proved effective for depolymerization of both neat PA‐6 and PA‐66 granules. After the reaction, water soluble products were formed, while some solid pieces of carpet remained. Photos of the carpet waste and obtained products can be seen in Figure S3, Supporting Information. The detailed reaction conditions and obtained results regarding the type of end‐products can be found in Table S2, Supporting Information.

#### 2.4.1 Characterization of the Commercial Carpet Waste and Obtained Solid End‐Products

**Figure 5:**
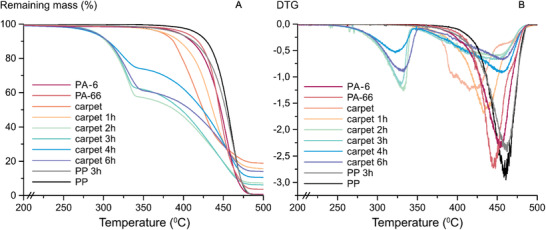
A) TGA analysis of neat carpet and microwave processed carpet after different microwave recycling times as well as reference PA‐6, PA‐66, and PP. B) First derivative of the TGA curves.

To monitor the recycling process and identify the solid end‐products, FTIR, DSC, energy‐dispersive X‐ray spectroscopy‐scanning electron microscopy (EDS‐SEM), and thermogravimetric analysis (TGA) were utilized. Neat PA‐6, neat PA‐66, neat PP, and microwave processed PP (PP‐3h) were also analyzed and compared to the recycled carpet. The PP‐3h was analyzed to see if the characteristic properties of PP change when subjected to the microwave process. The PP and PP‐3h both show the expected FTIR absorption bands, highlighted by grey at 2900, 1465, and 1376 cm^−1^. These bands represent the stretching transmittance of CH_2_ and CH_3_, CH_3_ and CH_2_.^[^ ^33^ ^]^ The spectra of the neat PA‐6, PA‐66, and carpet display typical absorption bands for PA, Figure 4A. The band at 3300 cm^−1^ belongs to the N—H stretching, the band at 2900 cm^−1^ belongs to CH_2_ stretching, and the two bands at 1700 and 1500 cm^−1^ belong to the carbonyl bond and the CH_2_ deformation, respectively.^[^ ^33^ ^]^ These results indicate that the neat carpet mainly has PA on the outside and PP backing on the inside. It is not possible from the FTIR spectra to determine if the PA in the carpet is PA‐6, PA‐66, or a mixture of both of them. However, three different melting peaks were observed in the DSC thermogram of the neat carpet, Figure 4B. These melting transitions match the melting temperatures of PP: 162 °C, PA‐6: 221 °C, and PA‐66: 262 °C, strongly indicating that the carpet consists of a mixture of PA‐6 and PA‐66 along with PP. The integration of the melting peaks in the DSC thermogram of the carpet and comparison with neat PA‐6, PA‐66, and PP, indicated that the ratio of the constituents was approximately 8% PP, 17% PA‐6, and 35% PA‐66. This approximation was calculated by utilizing the typical degrees of crystallinity as well as the theoretical degree of crystallinity for 100% crystalline PP, PA‐6, and PA‐66 with the help of Equations (2)–(4). The calculated composition corresponds well with a quite typical composition for a PA/PP carpet.^[^ ^14^ ^]^ However, the percentages could be somewhat underestimated, because the actual degree of crystallinity of the polymers in the carpet could be reduced due to, for example, blending of PA‐6 and PA‐66. The remaining material typically consists of adhesives and inorganic fillers like CaCO_3._^[^ ^29^ ^]^ The thermogravimetric analysis of neat carpets, indicates that the content of inorganics was approximately 20 wt% (**Figure** **5**). The degradation onset temperature for the carpet was also lower compared to neat PP, PA‐6, and PA‐66, which could indicate the presence of adhesives.

The absorption bands around 1700 and 3400 cm^−1^ became visible after 1 h of microwave processing. These absorption bands represent the end‐groups of solid PA oligomers that are formed during the hydrolysis reaction, Figure 4A, and they are no longer visible after 6 h of recycling, as the oligomers have hydrolysed into water soluble products during prolonged microwave processing. After 6 h of degradation, no PA is detectable by FTIR, indicating that all of the PA has been hydrolysed and only PP remains. This was further confirmed by the DSC trace of the remaining solid phases, see Figure 4B. The neat carpet has three different melting peaks whereas after 6 h, only one melting peak remains, which matches the melting peak of PP. Already after 1 h of microwave recycling, the melting transition of PA‐6 disappeared from the DSC thermogram. This agrees with the results from microwave recycling of neat PA‐6 and PA‐66, which showed that PA‐6 hydrolyses more easily than PA‐66.

After 2 h of microwave processing, the remaining solid phase started to degrade at a lower temperature during TGA analysis with a clear change in the slope compared to the material recovered after 1 h of microwave processing, see Figure 5. There can be several different explanations for this change. Some solid oligomeric species could be formed during the microwave processing, which was confirmed by FTIR. The solid oligomers subsequently degrade at lower temperature or the carpet could contain additives that degrade during microwave processing.

**Figure 6:**
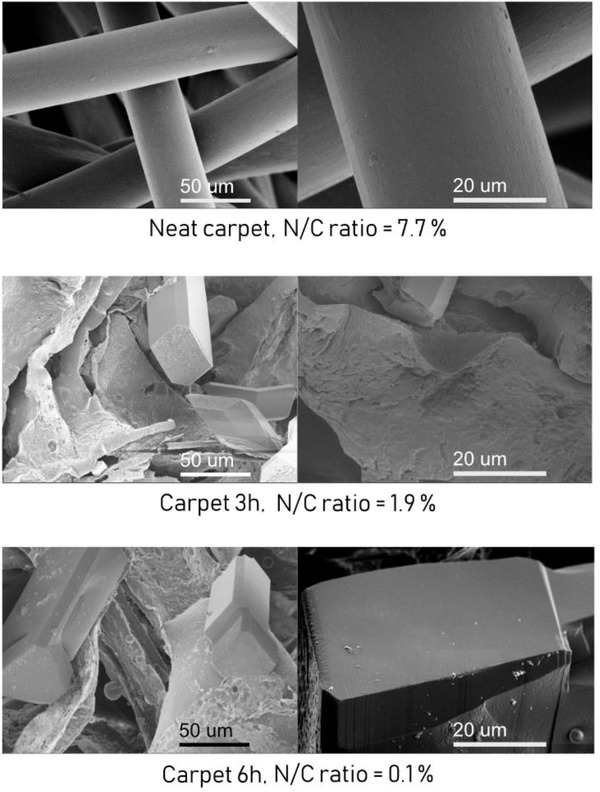
SEM images and EDS–SEM analysis of the remaining solid parts after microwave recycling of the commercial carpet.

Distinct fibers could be observed in the SEM images of the original neat carpet, **Figure** **6**. After 3 h of microwave processing, these fibers had disappeared and rod‐like structures could be observed instead. After 6 h of microwave processing, the rod‐like structures were still present. To evaluate the amount of nitrogen originating from the PA component left on the surface, EDS‐SEM was performed. The N/C ratio clearly decreased with increasing degradation time, indicating hydrolysis of PA‐6 and PA‐66. This further confirmed the results from FTIR and DSC analysis.

#### 2.4.2 PP Filaments Extruded from Blends of Virgin PP and Solid Carpet Residue

**Figure 7:**
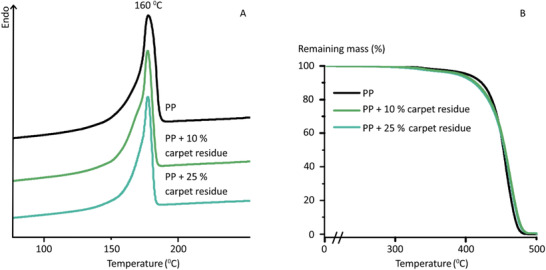
A) DSC thermogram of the extruded materials: neat PP and PP blends with 10 or 25 wt% of recycled carpet. B) TGA analysis of the same samples.

To verify that the remaining solid carpet residue after microwave assisted recycling could be utilized as a component in good quality PP products, it was directly used to make recycled filaments by mixing 10 or 25 wt% recycled carpet residue (6 h processing time) with virgin PP. Thermal analysis of the filaments by DSC and TGA, **Figure** **7** displays no differences between the filament containing only virgin PP and the filament containing 10 or 25 wt% carpet residue. This shows that the PP residue from recycling process does not significantly influence the quality, such as thermal stability of the PP products.

#### 2.4.3 Characterization of the Obtained Water‐Soluble Monomers

**Figure 8:**
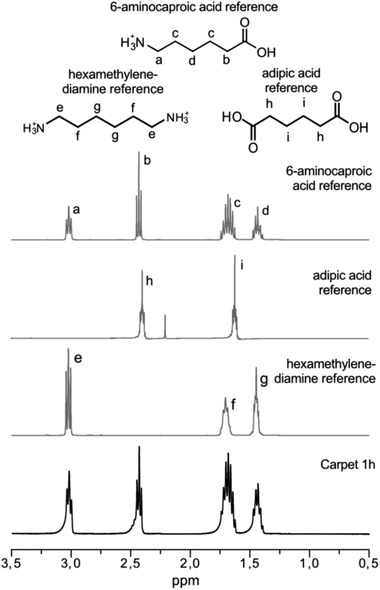
^1^H‐NMR spectra of the water‐soluble products formed during microwave recycling of commercial carpet. ^1^H‐NMR spectra of the expected monomers are included for comparison.

Hydrolysis of the PA part of the carpet was confirmed by the analysis of the water‐soluble monomers formed during microwave recycling. The ^1^H‐NMR spectra of the products formed after 1 h can be found in **Figure** **8**, while the spectra after 2 to 6 h of microwave processing can be found in Figure S4, Supporting Information. The signals match well with the signal of the expected monomers that is, protonated 6‐aminocaproic acid, adipic acid and protonated hexamethylenediamine. Furthermore, no other water‐soluble side‐products from colorants or adhesives could be detected, showing that the hydrolysis was to high degree selective toward PAs. In combination with the DSC analysis, the ^1^H‐NMR of the water‐soluble hydrolysis products further confirms that the PAs in the carpet are most likely PA‐6 and PA‐66. To purify the value‐added monomers, the mixture could be separated by vacuum distillation since the formed monomers have different boiling temperatures. After collection of the three different fractions, the purity could be checked by, for example, ^1^H‐NMR, thereafter the monomers could be directly used for repolymerization.^[^ ^13^ ^]^

The combined results from FTIR, DSC, TGA, EDS‐SEM, and ^1^H‐NMR clearly show that PA‐6 and PA‐66 in the commercial carpet were selectively hydrolyzed, leaving the PP part relatively intact.

## 3 Conclusion

An effective microwave‐assisted hydrothermal method to chemically recycle PA‐6 and PA‐66 from commercial multicomponent carpet was developed. PA‐6 and PA‐66 from the carpet were selectively hydrolysed into monomers, 6‐aminocaproic acid, adipic acid, and hexamethylene diamine. At the same time, the PP part was left relatively intact. The PP residue could be blended with virgin PP and extruded to good quality filaments with similar thermal properties to virgin PP. HCl was an effective catalyst for the reaction and the monomeric nature of the recovered hydrolysis products and the absence of side‐reactions under optimized conditions was confirmed by ^1^H‐NMR. The remaining solid part was analyzed after different reaction times by DSC, TGA, FTIR, and SEM‐EDS. The decreasing content of PA‐6 and PA‐66 in the carpet as a function of reaction time could be clearly observed, whereas PP was retained as solid residue. The developed process gives promise for the possibility to effectively recycle multicomponent materials to polymers and functional chemicals through selective hydrolysis of some of the components, contributing to a more circular economy.

## 4 Experimental Section

### Materials

The commercial carpet was a polyamide‐6 (PA‐6)/polyamide‐66 (PA‐66)/polypropylene (PP) rug from Kilands mattor. PA‐6 granules (crystallinity 21.3%) were from Akulon and PA‐66 granules (crystallinity 15.5%) from Technyl Polyamides. Polypropylene (crystallinity 41.1%) was purchased from Domolen. Hydrochloric acid (HCl, 37%), hexamethylenediamine (98%), 6‐aminocaproic acid (99%), and sulfuric acid (H_2_SO_4_, 95–98%) were purchased from Sigma–Aldrich. Phosphoric acid (H_3_PO_4_, 85–90%) and adipic acid (99%) were purchased from Fluka. Deuterium oxide (D_2_O, 99.96%) for NMR analysis and methanol (MeOH, 95.8%) were purchased from VWR. 1,1,1,3,3,3‐hexafluropropan‐2‐ol (HFIP) was obtained from Apollo and α‐cyano‐4‐hydroxycinnamic acid (>99%) from Fluka.

### Microwave‐Assisted Recycling of PA‐6 and PA‐66 Granules

PAs were processed in a microwave reactor in pure water (200 °C, 2 and 4 h) or MeOH (150 °C, 2 h).Three different catalysts, HCl (0.1 g mL^−1^, aqueous or MeOH), H_3_PO_4_ (0.1 g mL^−1^, aqueous), and H_2_SO_4_ (0.1 g mL^−1^, aqueous), were evaluated for their ability to accelerate the hydrolysis of PA‐6 and PA‐66. The reaction time was 2 h. Based on the assessment, aqueous HCl was chosen as a catalyst for further development of the recycling process. To further evaluate the acid catalyst, two different concentrations were investigated: 0.01 g mL^−1^ and 0.1 g mL^−1^. The run time was varied between 30 min and 3 h. The reaction conditions were the following: 0.5 g of granulated polymer sample was placed in the FlexiWave Teflon vial in 10 mL of HCl solution. The vial was placed in a FlexiWave microwave system from Milestone (Sorisole, Italy) with a maximum power of 1900 W. The temperature and pressure were followed by a probe inserted into a reference vessel. To reach the desired temperature, a 10 min ramp time was needed; thereafter, the temperature was held constant at 200 °C for the duration of the run. After the process, the vial was left to cool in a fume hood for at least 24 h. If solid material remained, it was separated from the liquid part by filtration and dried in a vacuum oven. The solvent was then removed by rotary evaporation to collect the water or MeOH soluble products, which were dried under vacuum before further analysis were performed.

### Microwave‐Assisted Recycling of Multicomponent Carpet Waste

The commercial carpet was cut into small squares and 0.5 g were placed in the Teflon vial together with 10 mL of 0.1 g mL^−1^ aqueous HCl. The reaction proceeded as above and the time was varied from 1 to 6 h. For comparison, neat PP was also subjected to the same procedure for 3 h (PP 3h). After the reaction, the solid part was separated from the liquid part by filtration and dried in a vacuum oven. The solvent was removed by rotary evaporation to collect the water‐soluble products, which were then dried under vacuum.

### Remaining Solid Material

The percentage of the remaining solid material after the microwave assisted reactions were calculated from the weight of the dried solid products and the initial weight of PA used for the reaction, by the following equation.

(1) $$\begin{array}{c} remaining\;solid\;material\;\%\;=\;\left(\frac{solid\;part\;\left(g\right)}{original\;polyamide\;\left(g\right)}\right)\times \;100 \end{array}$$

### Synthesis of New PA

The reactivity of the monomers obtained from the microwave‐assisted recycling of PA‐6 and PA‐66 was evaluated by synthesizing new PAs by step‐growth polymerization. The crude degradation products were used without further purification and placed in a round bottom flask equipped with a condenser under nitrogen atmosphere and stirring. The flask was placed in a heating mantel at 90 °C and the temperature was then gradually increased to 190 °C. Vacuum was applied when condensation drops were visible. The total reaction time was 6 h.

### Production of PP Filaments

The solid residue remaining after 6 h of microwave assisted recycling of carpet waste was mixed with neat PP (10 or 25 wt%) in a DSM micro compounder with coronating conical‐twin screws. The material was extruded for 2 min at 180 °C and 100 RPM.

### Characterization

*Nuclear Magnetic Resonance Spectroscopy (NMR)*: The chemical structure of the water‐soluble degradation products and the expected monomers in HCl solution as references, were analysed by a Bruker Avance DPX‐400 Nuclear Magnetic Resonance spectrometer (Billerica, MA, U.S.A.) operating at 400 MHz (^1^H NMR). The analysis was performed at room temperature using deuterium oxide (D_2_O) as a solvent.

### Matrix‐Assisted Laser Desorption Ionization‐Mass Spectrometry (MALDI‐MS)

The newly synthesized PAs, the original PA‐6 and PA‐66 granules, and the soluble products obtained from microwave assisted recycling of PA‐6 and PA‐66 at an HCl concentration of 0.01 g mL^−1^ were dissolved in HFIP (5 mg mL^−1^). α‐Cyano‐4‐hydroxycinnamic acid was used as matrix in HFIP (10 mg mL^−1^) and the matrix solution was mixed with sample at a volume ratio of 2:1. 1.5 microliters of that solution spotted on the MALDI target plate. All experiments were performed using a Bruker UltraFlex time‐of‐flight (TOF) mass spectrometer (Bruker Daltonic, Bremen, Germany) in a positive mode. The mass‐to‐charge (m/z) ratio range was 300–3000 with a reflector voltage of 26.3 kV and an accelerated voltage of 25 kV.

### Differential Scanning Calorimetry (DSC)

DSC analysis of the PAs, neat PP, PP‐3h, neat carpet recycled carpet, and extruded material was performed on a Metter Toledo DSC 820 (Stockholm, Sweden). Approximately 5 mg of each sample was enclosed into standard 100 µL aluminium cups. Samples were heated under a nitrogen atmosphere from 25 °C to 300 °C at a rate of 10 °C min^−1^, held at 300 °C for 10 min and then cooled to −30 °C at a rate of 10 °C min^−1^, held at −30 °C for 10 min and then finally heated to 300 °C at a rate of 10 °C min^−1^. Duplicate samples were analyzed. The composition of the neat carpet was approximated with the help of theoretical *ΔH*_0_ values for 100% crystalline PP, PA‐6, and PA‐66 by utilizing following equations. PP is used as an example:

(2) $$\begin{array}{c} X_{c,\;\text{neat}\;\text{PP}\;}\;\left(\%\right)=\frac{\Delta H_{m}\left(\frac{J}{g}\right)}{\Delta H_{0}\left(\frac{J}{g}\right)}\;\;\times \;100 \end{array}$$

(3) $$\begin{array}{c} X_{c,\text{carpet}\;\text{PP}\;}\;\left(\%\right)=\frac{\Delta H_{m}\left(\frac{J}{g}\right)}{\Delta H_{0}\left(\frac{J}{g}\right)}\;\;\times \;100 \end{array}$$

(4) $$\begin{array}{c} ratio\;\left(\%\right)\;=\;\frac{X_{c,\text{carpet}\;\text{PP}}\left(\%\right)}{X_{c,\;\text{neat}\;\text{PP}\;}\left(\%\right)}\;\times \;100 \end{array}$$

### Thermogravimetric Analysis (TGA)

Mettler–Toledo TGA/SDTA 851e was utilized for TGA measurements of neat carpet, neat PAs, and PP, PP‐3h, recycled carpet and extruded material. ≈5 mg of each sample was placed into a 70 µL alumina cup. The samples were heated at a rate of 10 °C min^−1^ from 30 °C to 300 °C under 80 mL min^−1^ nitrogen flow. Double samples were analyzed for each material.

### Fourier‐Transform Infrared Spectroscopy (FTIR)

The analysis of neat PP, PP‐3h, neat PAs, neat carpet, and recycled carpet were conducted on a Perkin Elmer Spectrum 100 equipped with an attenuated total reflectance (ATR) accessory (golden gate) from Graseby, U.K. with 16 scans from 4000 to 600 cm^−1^ through a resolution of 8 cm^−1^. The software was PerkinElmer, Inc. version 10.5.1.

### Scanning Electron Microscopy (SEM)

Ultrahigh‐resolution FE‐SEM (Hitachi S‐4800) was utilized to study the surface morphology of the neat carpet and recycled carpet. The samples were coated with a 20 nm layer by a Pt/Pd source. Energy dispersive X‐ray spectroscopy (EDS) spectra were acquired on the same Hitachi S‐4800 SEM, equipped with an Oxford Instruments X‐MaxN 80 EDS at a voltage of 20 kV. The elements were acquired by an average of 10 point‐ID measurements. The N/C ratio was estimated by dividing the average wt% of nitrogen with the average wt% of carbon.

## Conflict of Interest

The authors declare no conflict of interest.

## Supporting information

Supporting Information

## Data Availability

The data that support the findings of this study are available from the corresponding author upon reasonable request.

## References

[1] A. F. Richards , in Synthetic Fibres: Nylon, Polyester, Acrylic, Polyolefin, (Ed: J. E. McIntyre ), Elsevier Ltd., Amsterdam, the Netherlands 2004, pp. 20–94.

[2] Man‐Made Fibers Continue To Grow | Textile World, https://www.textileworld.com/textile-world/fiber-world/2015/02/man-made-fibers-continue-to-grow/ (accessed: April 2020).

[3] S. R. Shukla , A. M. Harad , D. Mahato , J. Appl. Polym. Sci. 2006, 100, 186.

[4] Transition to a toxic‐free circular economy in the carpet industry still a distant reality – Zero Waste Europe.

[5] A. M. Nemade , S. Mishra , V. S. Zope , J. Polym. Environ. 2011, 19, 110.

[6] D. B. Patil , S. V. Madhamshettiwar , J. Appl. Chem. 2014, 2014, 286709.

[7] M. Xanthos , A. Patel , S. Dey , S. S. Dagli , C. Jacob , T. J. Nosker , R. W. Renfree , Adv. Polym. Technol. 1994, 13, 231.

[8] A. Sotayo , S. Green , G. Turvey , Environ. Technol. Innovation 2015, 3, 97.

[9] D. Lazarevic , E. Aoustin , N. Buclet , N. Brandt , Resour., Conserv. Recycl. 2010, 55, 246.

[10] J. Datta , P. Kopczyńska , Crit. Rev. Environ. Sci. Technol. 2016, 46, 905.

[11] T. Keijer , V. Bakker , J. C. Slootweg , Nat. Chem. 2019, 11, 190. 30792512 10.1038/s41557-019-0226-9

[12] EUR‐Lex –52018DC0028 – EN – EUR‐Lex, https://eur-lex.europa.eu/legal-content/EN/TXT/?qid=1516265440535&uri=COM:2018:28:FIN (accessed: March 2020).

[13] C. Mihut , D. K. Captain , F. Gadala‐Maria , M. D. Amiridis , Polym. Eng. Sci. 2001, 41, 1457.

[14] A. T. Griffith , Y. Park , C. B. Roberts , Polym. ‐ Plast. Technol. Eng. 2008, 38, 411.

[15] A. Carné Sánchez , S. R. Collinson , Eur. Polym. J. 2011, 47, 1970.

[16] H. R. Kricheldorf , M. Al Masri , G. Schwarz , Macromolecules 2003, 36, 8648.

[17] E. Bäckström , K. Odelius , M. Hakkarainen , ACS Sustainable Chem. Eng. 2019, 7, 11004.

[18] J. Payne , P. McKeown , M. D. Jones , Polym. Degrad. Stab. 2019, 165, 170.

[19] S. Hongthong , S. Raikova , H. S. Leese , C. J. Chuck , Waste Manage. 2020, 102, 351. 10.1016/j.wasman.2019.11.003 31726315

[20] A. Kamimura , K. Kaiso , S. Suzuki , Y. Oishi , Y. Ohara , T. Sugimoto , K. Kashiwagi , M. Yoshimoto , Green Chem. 2011, 13, 2055.

[21] U. Klun , A. Kržan , Polymer 2000, 41, 4361.

[22] U. Klun , S. Andrenšek , A. Kržan , Polymer 2001, 42, 7095.

[23] U. Češarek , D. Pahovnik , E. Žagar , ACS Sustainable Chem. Eng. 2020, 8, 16274. 33194457 10.1021/acssuschemeng.0c05706 PMC7662757

[24] X. Yang , K. Odelius , M. Hakkarainen , ACS Sustainable Chem. Eng. 2014, 2, 2198.

[25] D. Wu , M. Hakkarainen , ACS Sustainable Chem. Eng. 2014, 2, 2172.

[26] E. Bäckström , K. Odelius , M. Hakkarainen , Ind. Eng. Chem. Res. 2017, 56, 14814.

[27] K. H. Adolfsson , C. Lin , M. Hakkarainen , ACS Sustainable Chem. Eng. 2018, 6, 11105.

[28] H. Bockhorn , S. Donner , M. Gernsbeck , A. Hornung , U. Hornung , J. Anal. Appl. Pyrolysis 2001, 58–59, 79.

[29] M. Braun , A. B. Levy , S. Sifniades , Polym. ‐ Plast. Technol. Eng. 2008, 38, 471.

[30] H. Choi , K. C. Eun , K. Y. Eun , S. Jang , R. P. Chan , Bull. Korean Chem. Soc. 2007, 28, 2354.

[31] A.‐C. Albertsson , U. Edlund , K. Odelius , Polymerteknologi: Makromolekylär Design, Kungl. Tekniska högskolan, Stockholm, Sweden 2012.

[32] J. Chen , Z. Li , L. Jin , P. Ni , G. Liu , H. He , J. Zhang , J. Dong , R. Ruan , J. Mater. Cycles Waste Manage. 2010, 12, 321.

[33] E. Kang , M. Kim , J. S. Oh , D. Wha Park , S. E. Shim , Macromol. Res. 2012, 20, 372.

