# Supplementary material for: Microwave Assisted Selective Hydrolysis of Polyamides from Multicomponent Carpet Waste
Source: Glob Chall. 2021 May 5;5(7):2000119. doi: 10.1002/gch2.202000119 (PMC8272014; doi:10.1002/gch2.202000119)
Supplement: Supplementary file 1 — Supporting Information [file GCH2-5-2000119-s001.pdf]

© 2021 Wiley-VCH GmbH

Global Challenges Open Access

Supporting Information

for *Global Challenges,* DOI: 10.1002/gch2.202000119

Microwave Assisted Selective Hydrolysis of Polyamides from Multicomponent Carpet Waste

*Eva Bäckström, Karin Odelius, and Minna Hakkarainen**

Microwave assisted selective hydrolysis of polyamides from multicomponent carpet waste

*Eva Bäckström, Karin Odelius, and Minna Hakkarainen**

Table S1. Summary of reaction conditions and type of products obtained after microwave recycling of PA-6 and PA-66 in water for 2 and 4 h at 200 ºC.

Staring material	time (h)	H_2O	Solid products remaining	Water soluble products
PA-6	2	only	yes	no
PA-6	4	only	yes	no
PA-66	2	only	yes	no
PA-66	4	only	yes	no

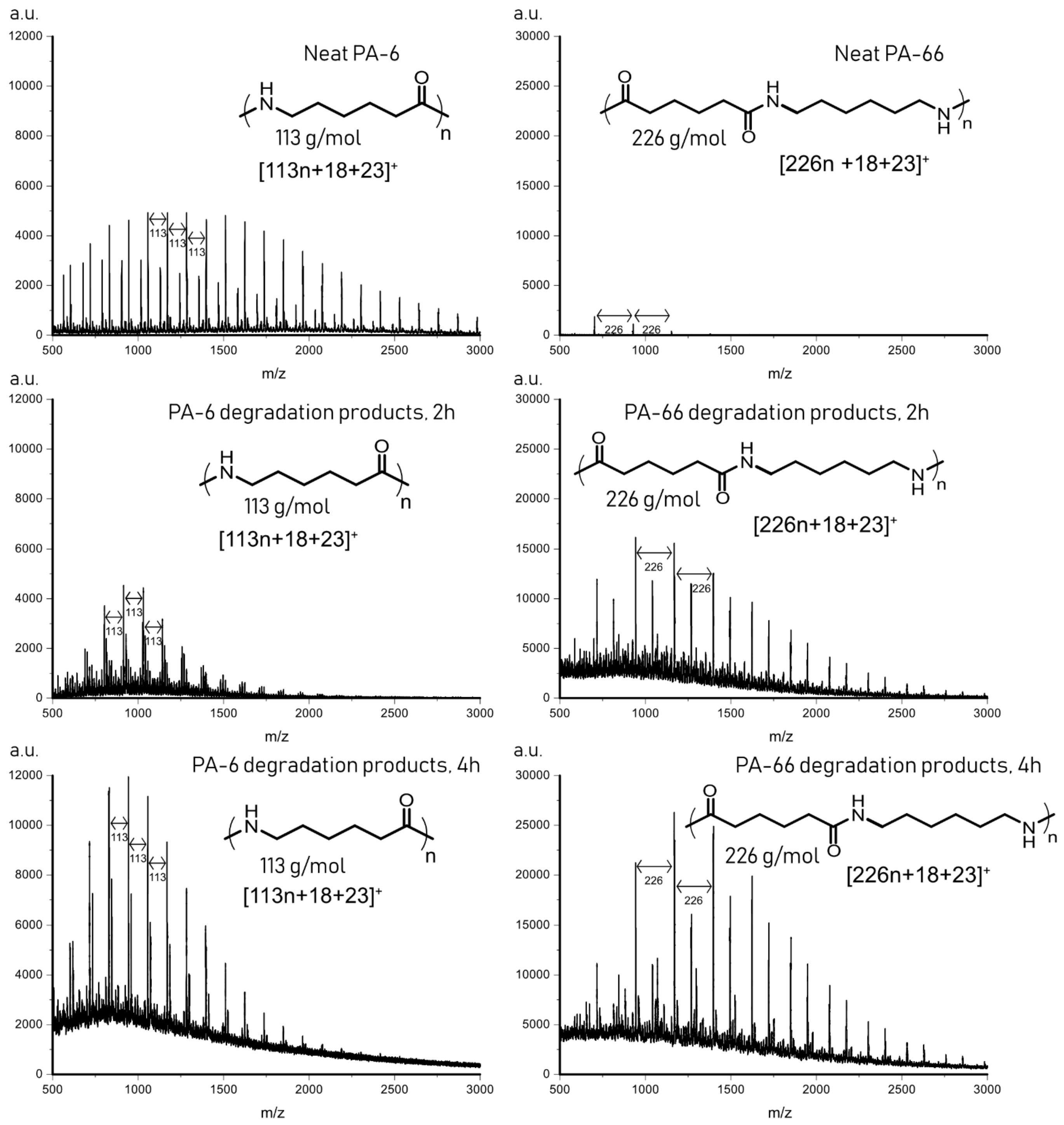

Figure S1. MALDI-MS mass spectra of the oligomers formed during microwave processing of PA-6 and PA-66 in water for 2 and 4 h at 200 ºC. In the m/z equation 113n and 226n correspond to the molecular weights of the repeating units for PA-6 and PA-66, 18 corresponds to the molecular weight of the end groups, -H and -OH, and 23 is the molecular weight of Na.

Synthesis of new polyamides. To verify that the monomers obtained after microwave-assisted recycling of PA-6 and PA-66 could be used to produce new PAs, they were re-polymerized by step-growth polymerization directly from the crude degradation product mixture from microwave-assisted recycling (2 h at 200 ºC). The concentration of HCl catalyst was 0.1 g/ml. MALDI-MS analysis revealed oligomeric polyamides as hydrogen or sodium adducts corresponding to m/z = 113n + 18+ 1 for PA-6 and m/z = 226n+ 18 + 23 for PA-66. 113 and 226 correspond to the molecular weights of the repeating units of PA-6 and PA-66. 18 is the molecular weight of the end-groups (-H and -OH) and 1 and 23 correspond to H-or Na-adducts of the oligomers. The highest intensity peaks displayed a m/z value around 1000-1200.

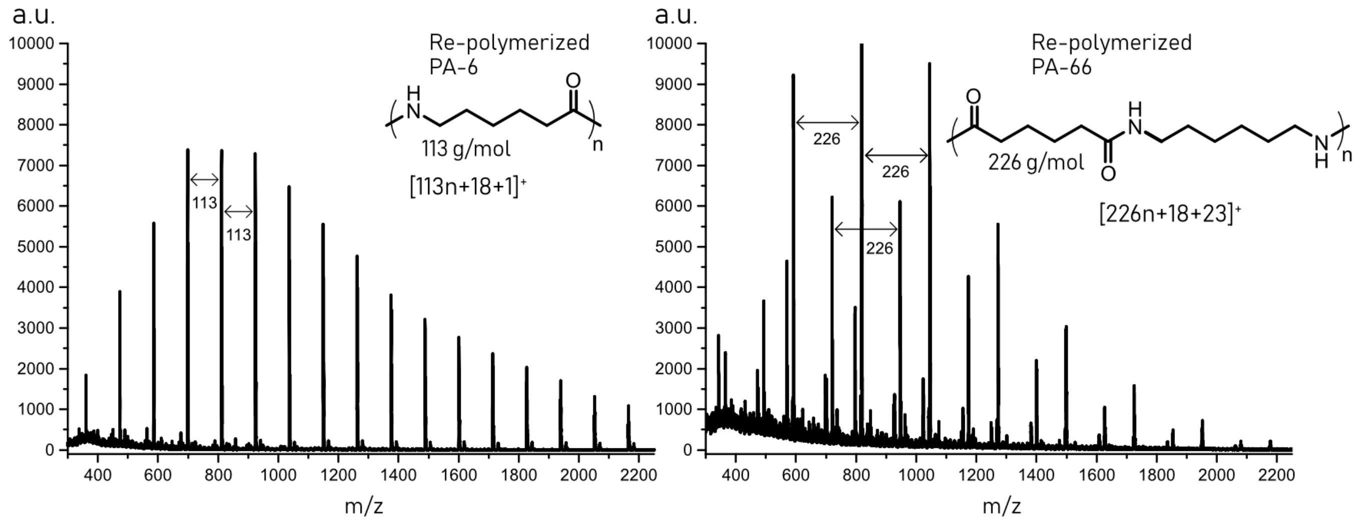

Figure S2. MALDI-MS mass spectra of the new, synthesized PAs.

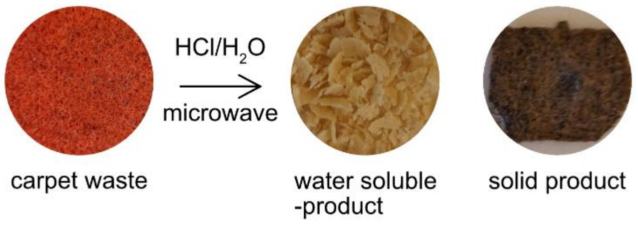

Figure S3. Photo of the commercial carpet, the water-soluble products (after evaporation of water) and the solid product filtrated from the aqueous solution after 6 h of microwave heating.

Table S2. Summary of the reaction conditions and type of products obtained after microwave recycling of carpet waste.

Staring material	time (h)	C HCl aq (g/ml)	Remaining solid material (%)	Water soluble products
carpet	1	0.1	29	yes, monomeric
carpet	2	0.1	26	yes, monomeric
carpet	3	0.1	28	yes, monomeric
carpet	4	0.1	28	yes, monomeric
carpet	6	0.1	17	yes, monomeric

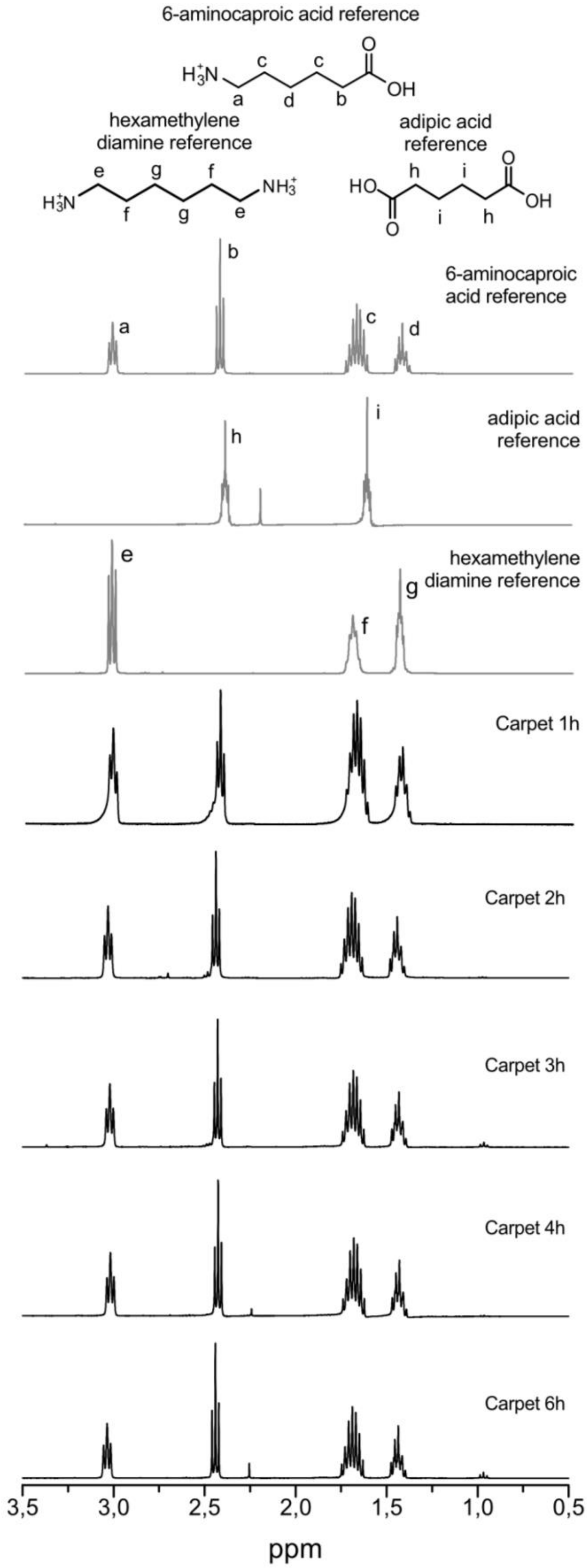
6-aminocaproic acid reference
hexamethylene diamine reference
adipic acid reference
6-aminocaproic acid reference
adipic acid reference
hexamethylene diamine reference
Carpet 1h
Carpet 2h
Carpet 3h
Carpet 4h
Carpet 6h
3,5
3,0
2,5
2,0
1,5
1,0
0,5
ppm

Figure S4. ^{1}H-NMR spectra of the water-soluble products formed during microwave recycling of model carpet waste, 1-6 h. ^{1}H-NMR spectra of the expected monomers are included for comparison.
